# The interaction between cucurbit[8]uril and baicalein and the effect on baicalein properties

**DOI:** 10.3762/bjoc.16.9

**Published:** 2020-01-10

**Authors:** Xiaodong Zhang, Jun Xie, Zhiling Xu, Zhu Tao, Qianjun Zhang

**Affiliations:** 1 Key Laboratory of Macrocyclic and Supramolecular Chemistry of Guizhou Province, Guizhou University, Guiyang 550025, China

**Keywords:** baicalein, cucurbit[8]uril, host–guest interaction, inclusion complex, properties

## Abstract

The host–guest interactions between baicalein (BALE) and cucurbit[8]uril (Q[8]) and the corresponding properties of the inclusion complex were studied using ^1^H NMR, IR and UV–vis spectroscopy and DTA. The results showed that BALE forms an inclusion compound (1:1) with Q[8], and the properties of baicalein are changed by cucurbit[8]uril.

## Introduction

**Figure 1:**
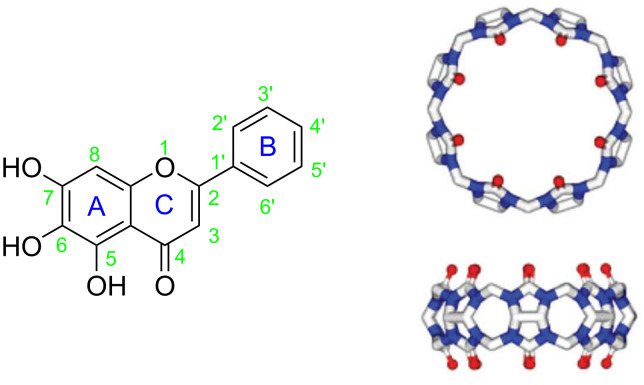
Chemical structures of baicalein (left), cucurbit[8]uril (right).

Baicalein (5,6,7-trihydroxyflavonoid) has a molecular formula of C_15_H_10_O_5_ (BALE, Figure 1) and is a natural flavonoid found in the roots of *Scutellaria baicalensis Georgi* [1]. The compound displays pharmacological activity such as antimicrobial, anti-inflammatory, anti-allergic, antispasmodic, diuretic and anticancer [2–24]. For example, baicalein can play an antitumor effect on lung cancer by inducing cell apoptosis, blocking cell cycle and inhibiting metastasis of lung cancer, but it has a strong activity to eliminate superoxide radicals in cell-free systems [25]. It is also considered as an anti-inflammatory agent [26–27] that generally protects against oxidative stress [28], more specifically in cardiac cells [29], and in cisplatin-induced acute kidney injury [30]. However, baicalein contains three phenolic hydroxy groups, which are easily oxidized to the quinone derivative and appear green, therefore, it has limited use in pharmaceuticals on account of its poor aqueous solubility and stability [31].

The cucurbit[*n*]urils (Q[*n*]s *n* = 5–8, 10, …) are macrocyclic hosts with a hydrophobic rigid cavity [32] (Figure 1). cucurbit[*n*]urils have a unique combination of properties including rigid highly symmetric structures, relatively large hydrophobic cavities and high thermal and chemical stability [33–34]. Cucurbit[*n*]urils are a type of macrocyclic drug carrier similar to macrocyclic compounds such as calixarenes, crown ethers and cyclodextrins, which can be used to form a stable inclusion complex with the drug, and improving the bioavailability of the drug [34–35]. Herein, we describe the results of the investigations of host–guest interactions between BALE and Q[8] in an aqueous solution using ^1^H NMR, UV–vis and IR spectroscopy, and DTA. The properties of the BALE–Q[8] inclusion complex, such as stability, solubility, in vitro antioxidant activity and release performance were studied by means of UV–vis spectroscopy.

## Results and Discussion

### Host–guest interactions

**Figure 2:**
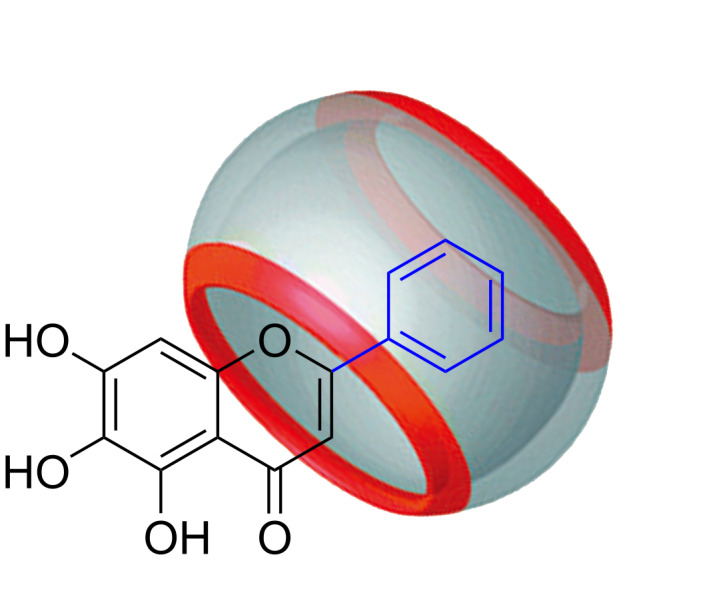
The possible interaction model for Q[8] and baicalein.

**Figure 3:**
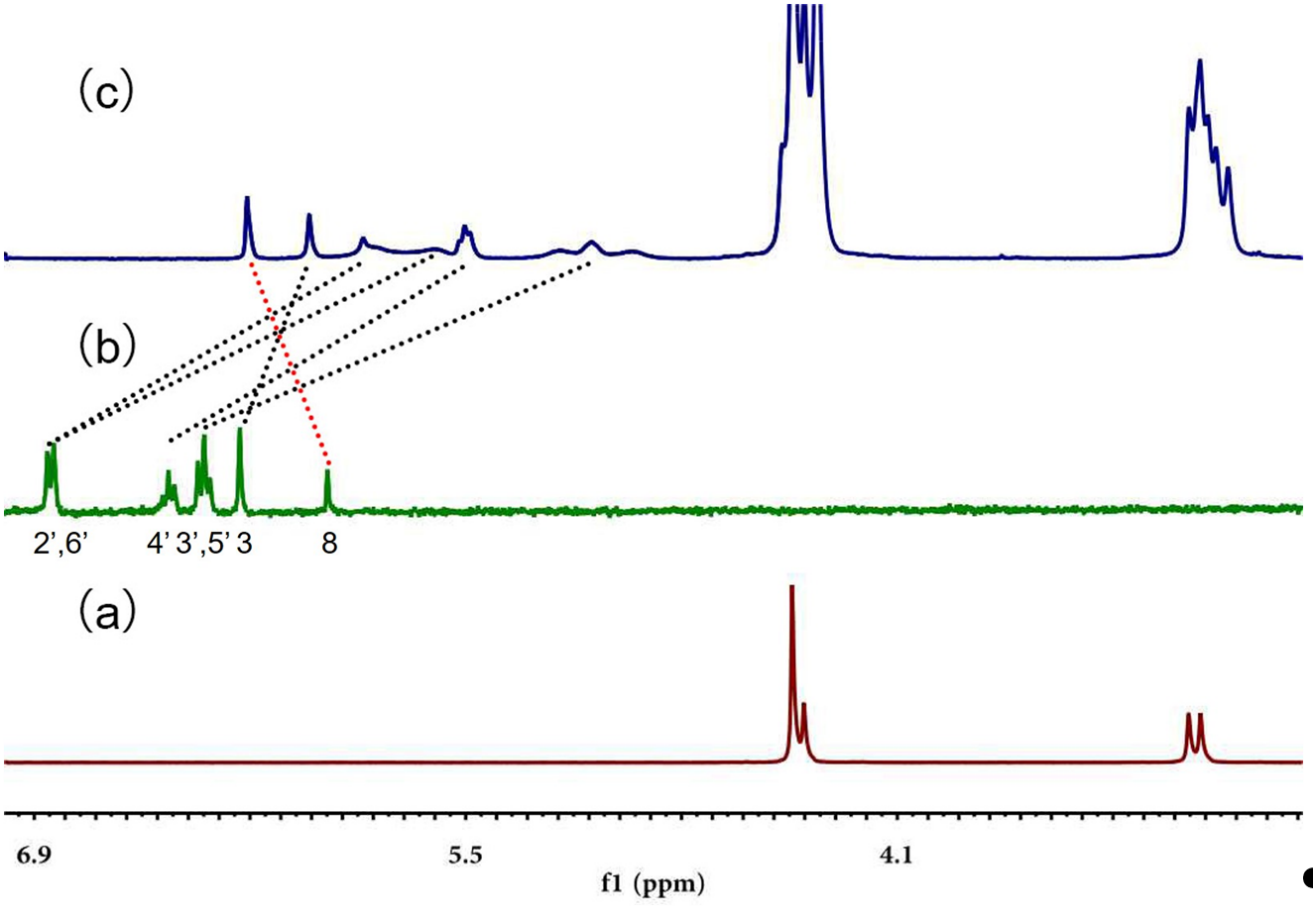
^1^H NMR spectra (400 MHz) of Q[8] (a), baicalein (b) and BALE–Q[8] (1:1) (c) recorded in DCl.

Q[8] and BALE in the host–guest interaction packing and pattern are shown in Figure 2. ^1^H NMR spectroscopy is usually one of the most effective methods to characterize host–guest interactions and can be used to deduce the mode of action of the cucurbit[*n*]uril–guest according to the chemical shift of the proton resonances. The size of the Q[8] cavity is larger than that of the guest (BALE), therefore the frequency of dissociation and inclusion of BALE by Q[8] was faster than the working frequency of the NMR instrument [36–40]. The ^1^H NMR spectra of BALE–Q[8] show only averaged proton resonances. Chemical shift changes of certain proton resonances of the guest or host upon increasing or decreasing the equivalents of the guest or host can be used to study the host–guest interactions. Figure 3 shows the ^1^H NMR spectra of BALE in the absence (b) and in the presence of 1.0 equiv of Q[8] (c). After adding Q[8], the H3, H2', H3', H4', H5' and H6' protons of the guest BALE underwent an upfield shift, but H8 exhibited a downfield shift (Table 1). The ^1^H,^1^H-NOESY spectrum reveals the close relationship between the intramolecular protons in space (Supporting Information File 1, Figure S1). This suggested that Q[8] included cycle B and part of cycle C of BALE into its cavity.

**Table 1:** Changes in the ^1^H NMR chemical shifts.

^1^H nucleus	Δδ/ppm	^1^H,^1^H-NOESY

2′,6′	1.25 1.02	33
4′	0.96	3′, 5′
3′,5′	1.26	4′
3	0.22	2′, 6′
8	−0.26	

**Figure 4:**
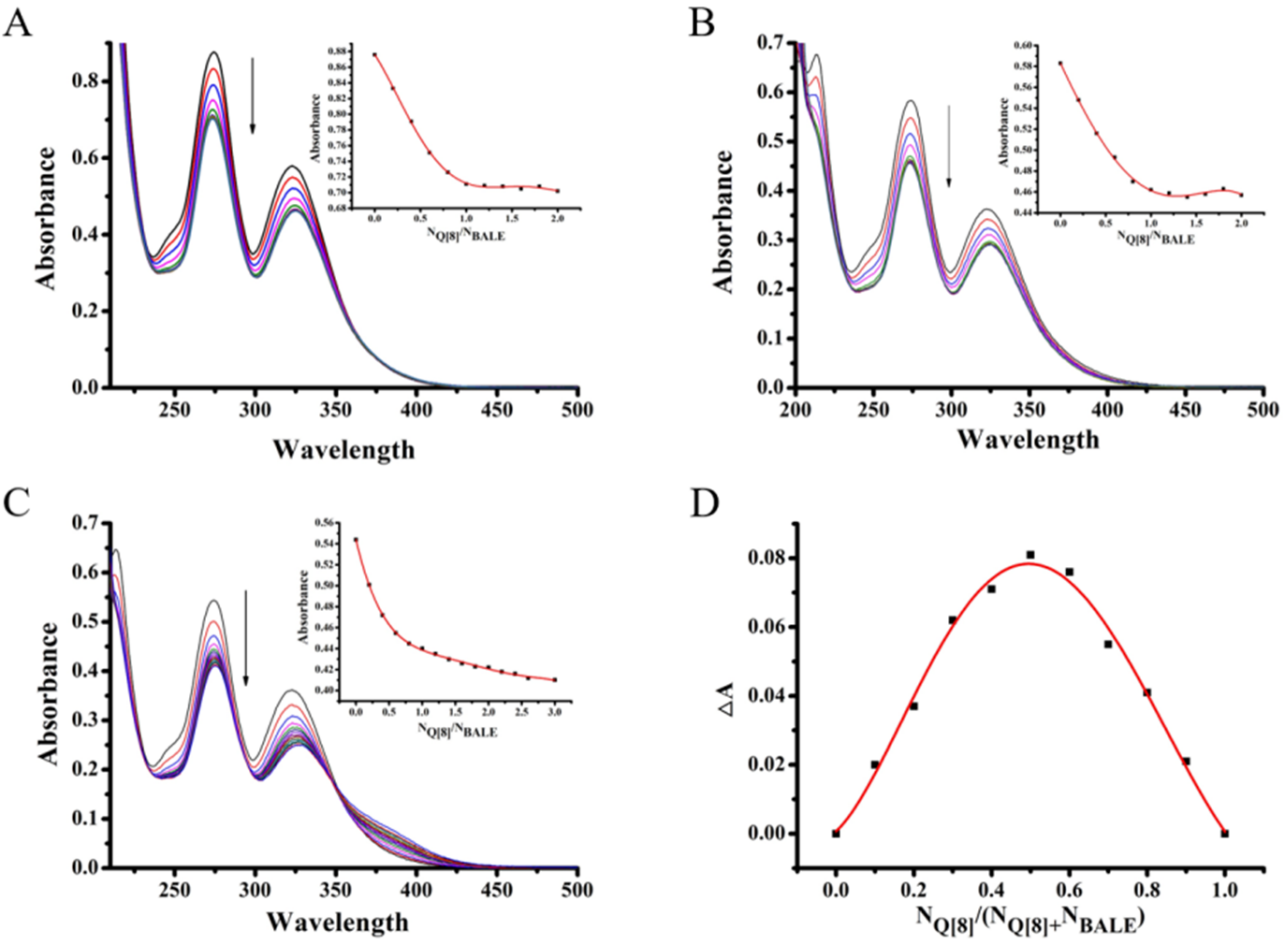
The absorption spectra of BALE upon the addition of Q[8] under different conditions [10 mol·L^−1^ HCl (A), 10^−2^ mol·L^−1^ HCl (B) and water(C)]; Job’s plot of A vs N_Q_[8]/N_Q_[8] + N_BALE_ (D).

To quantitatively determine the ratio of the host–guest inclusion complexes formed from Q[8] and the guest, the UV spectra of different aqueous solutions containing a fixed concentration of the guest (20 μmol·L^−1^) and variable concentrations of Q[8] were recorded (Figure 4A–C). Figure 4 shows the changes in the UV–vis spectra of BALE upon the gradual addition of Q[8]. The maximum absorption wavelength of BALE was at 270 nm and the host showed no absorbance in the range of >210 nm. The absorption spectra of the guest exhibited a progressively higher absorbance with a slight red shift as the molar ratio of N_Q_[8]/N_BALE_ increased. The absorbance (Figure 4A–C) vs molar ratio of the host Q[8] and guest (N_Q_[8]/N_BALE_) was fitted to a 1:1 binding model to afford the association constants (*K*) [2.15 × 10^7^ L·mol^−1^ (10 mol·L^−1^ HCl), 3.59 × 10^7^ L·mol^−1^ (10^-2^ mol·L^−1^ HCl) and 3.4 × 10^5^ L·mol^−1^ (neutral water)]. When compared with the change observed in the spectra recorded in 10 mol·L^−1^ HCl, 10^−2^ mol·L^−1^ HCl and water, the same interactive model was observed. The existence of clear isosbestic points and the Job’s plot (Figure 4D) supported the formation of a 1:1 host–guest inclusion complex. Q[8] and BALE in the host–guest interaction package and pattern are shown in Figure 2.

The mass spectrum of the BALE–Q[8] inclusion complex (Supporting Information File 1, Figure S2) features the parent-ion peak at *m*/*z* 1599.4534 (M^+^), supporting the formation of a 1:1 inclusion complex between BALE and Q[8].

**Figure 5:**
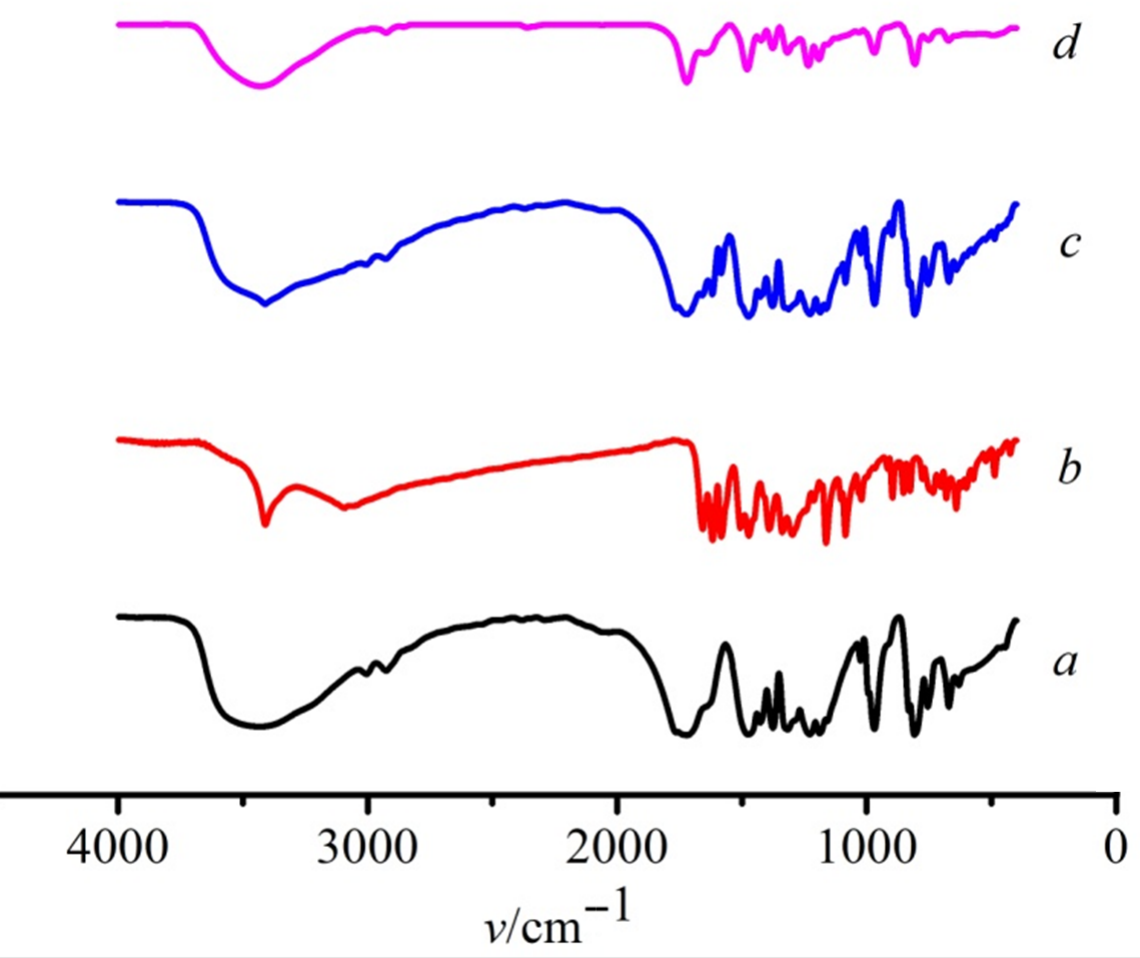
IR spectra of Q[8] (a), BALE (b), a physical mixture of Q[8]-BALE (N_Q_[8]/N_BALE_ = 1:1) (c) and the BALE–Q[8] inclusion complex (d).

The IR spectra recorded for the interaction of Q[8] with BALE are shown in Figure 5. It shows the IR spectra of Q[8] (a), BALE (b), a physical mixture of Q[8] and BALE (N_(Q_[8]_)_/N_(BALE)_ = 1:1) (c) and the BALE–Q[8] inclusion complex (d). Spectrum (c) was a simple superposition of BALE (a) and Q[8] (b), there was no interaction observed in the physical mixture. When compared to spectra (c) and (d), there were two absorption peaks observed at ≈3030 cm^−1^ corresponding to the stretching vibration of the two aromatic rings in BALE, but the complexes had only one absorption peak. In the range of 1650–1550 cm^−1^ were two absorption peaks for the two benzene ring stretching vibrations of BALE and the peak disappearance observed in the inclusion complex, which may be caused by Q[8]. In addition, there were differences in the fingerprint region of the benzene ring. In spectrum (d), the peaks at 704, 778 and 898 cm^−1^ for the guest disappeared due to the inclusion of Q[8] and the molecular microenvironment of BALE being changed.

**Figure 6:**
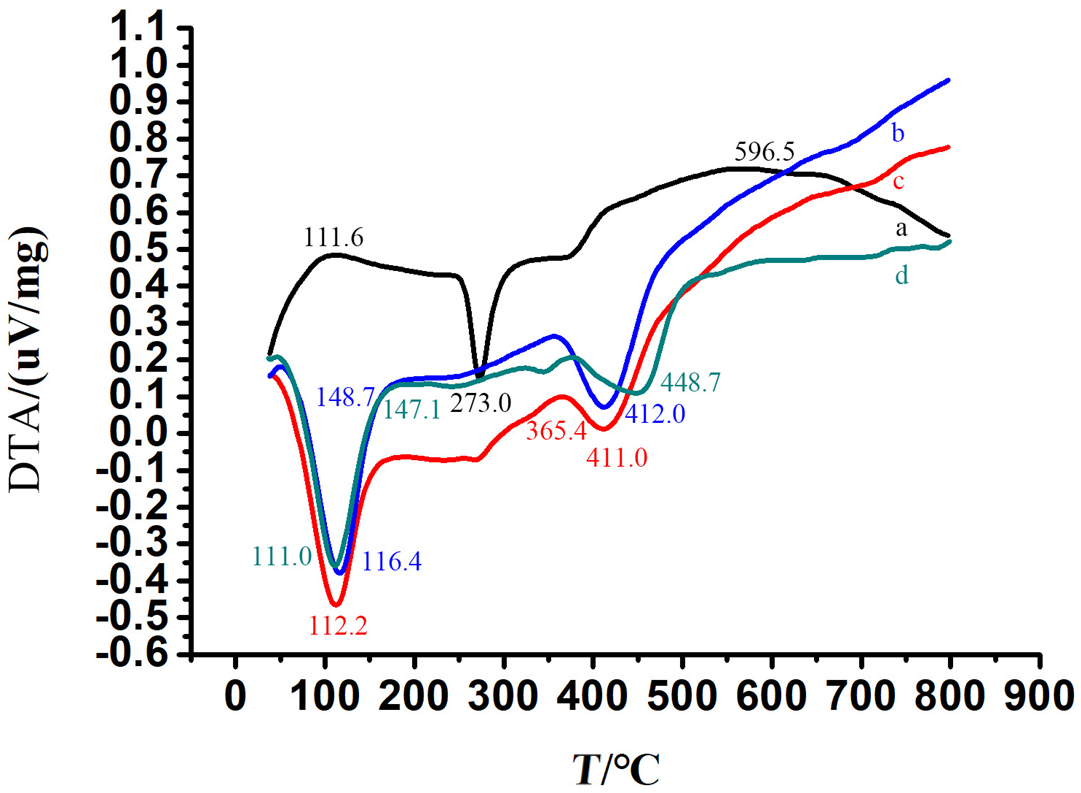
DTA spectra of BALE (a), Q[8] (b), a physical mixture Q[8]-BALE (N_Q_[8]/N_BALE_ = 1:1) (c) and the BALE–Q[8] inclusion complex (d).

From DTA (Figure 6), we can also see that BALE and Q[8] interacted with each other. Q[8] (Figure 6b) has a broad endothermic peak at 412.0 °C displaying its amorphous nature. Crystalline BALE (Figure 6a) has a sharp melting endothermic peak at 273.0 °C. The physical mixture of Q[8] and BALE (Figure 6c) presents the melting point and endothermic peak of BALE and Q[8] at 273.0 °C and 411.0 °C, respectively, but the BALE–Q[8] inclusion complex had an obvious melting point at 448.7 °C and the melting point and endothermic peaks of BALE and Q[8] disappeared. The results showed that a new material appeared, which improved the thermal stability of BALE (Figure 6d).

### The effect of cucurbit[8]uril on baicalein properties

#### Stability study

**Figure 7:**
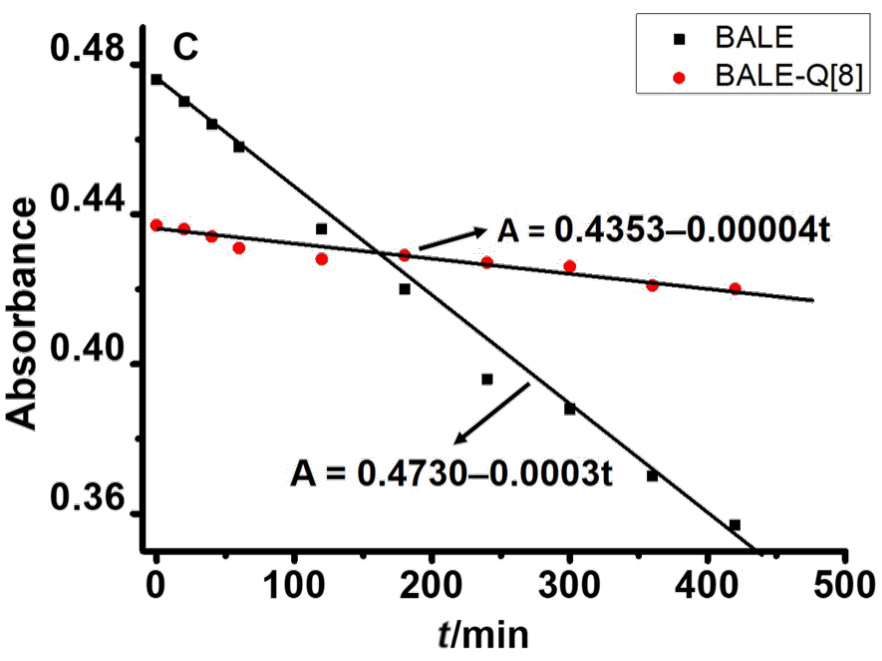
The stability curve of UV–vis absorption obtained for an isoconcentration of BALE and the BALE–Q[8] inclusion complex with time.

BALE has three phenolic hydroxy groups, which are easily oxidized to a quinone-type structure. The time-resolved UV–vis absorption spectra of BALE showed that the stability of BALE in water was poor in the absence of Q[8], whereas the stability of BALE was greatly improved in the presence of Q[8]. At the same concentration of BALE and the Q[8]–BALE complex, the free BALE absorption value, after 6 h, decreased by 0.1 or less (Figure 7) and the decomposition curve equation was *A* = 0.4730 – 0.0003t. However, the UV absorption intensity of the inclusion complex remained basically unchanged, very stable and the decomposition curve equation was A = 0.4353 − 0.00004t. Therefore, the stability of the BALE–Q[8] inclusion complex in the same solvent was 7.5 times higher than that of BALE.

#### Solubilization studies

**Figure 8:**
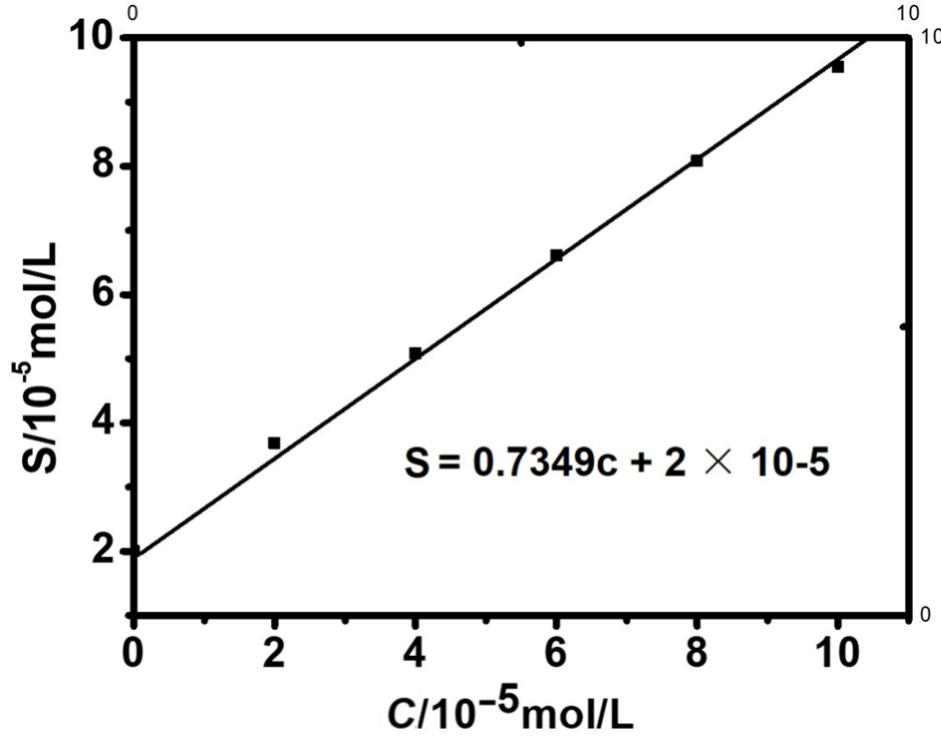
The phase solubility graph obtained for BALE in Q[8] at λ = 270 nm.

Solubilization studies were performed according to [41]. When compared with the unbound BALE solubility (Figure 8), the concentration in the presence of Q[8] reached at least 1.319 × 10^−5^ mol·L^−1^, which was a 6.98-fold increase for BALE over that in neutral water (1.889 × 10^−6^ mol·L^−1^), indicating that Q[8] had a significant solubilization effect on BALE. The solubility curve equation was *S* = 0.7349*c* + 2 × 10^−5^ (R = 0.999) and *S*_0_ = 2 × 10^−5^ mol·L^−1^. When *C*_Q_[8] = 10^−4^ mol·L^−1^, the solubility of BALE increased 4.67-fold.

#### Antioxidation studies

**Figure 9:**
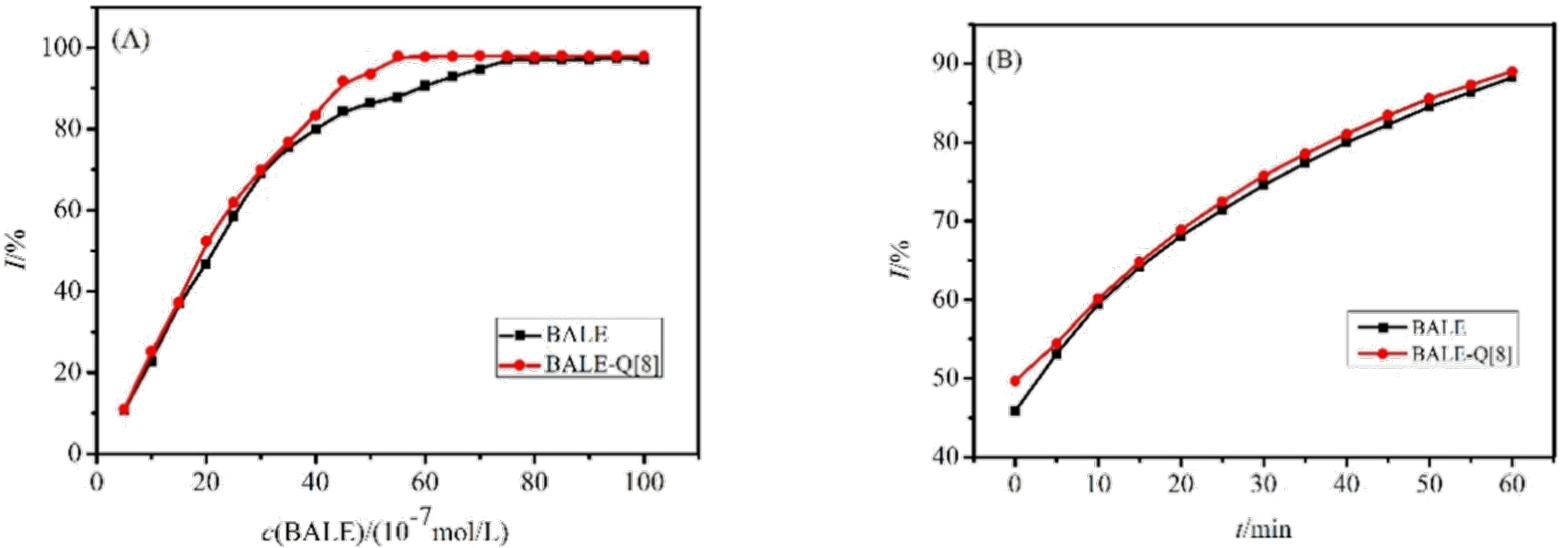
The clearance rate curve (A) and clearance time curve (B) of ABTS^+·^ upon increasing the concentration of BALE and the BALE–Q[8] inclusion complex.

Antioxidation studies were performed according to [42]. BALE has a strong ability to eliminate ABTS^+·^ (see ABTS radical-scavenging assay in Supporting Information File 1). If the BALE–Q[8] complex significantly reduces its antioxidant activity, this will affect the medicinal value of BALE. Figure 9 shows that Q[8] has no significant influence on the antioxidant activity of BALE. At 0.0005–0.004 mmol·L^−1^, BALE exhibited a linear increasing ability to eliminate ABTS^+·^ and when the concentration was >0.007 mmol·L^−1^, the eliminating ability of BALE was stabilized (Figure 9A). With an increase in concentration, the BALE–Q[8] complex also showed a linear increasing ability to eliminate ABTS^+·^ and when the concentration was >0.007 mmol·L^−1^, the eliminating ability was stabilized, too (Figure 9A). We also found that the ability to eliminate ABTS^+·^ of BALE and the BALE–Q[8] complex was proportional to the function of time (Figure 9B). The IC_50_ was calculated using the clearance rate curve after 30 min, which showed the IC_50_ of BALE was 1.87 × 10^–6^ mol·L^−1^ and the IC_50_ of the BALE–Q[8] complex was 2.14 × 10^–6^ mol·L^−1^. The ability of the BALE–Q[8] complex to eliminate ABTS^+^ is slightly stronger than that of BALE.

#### In vitro release studies

**Figure 10:**
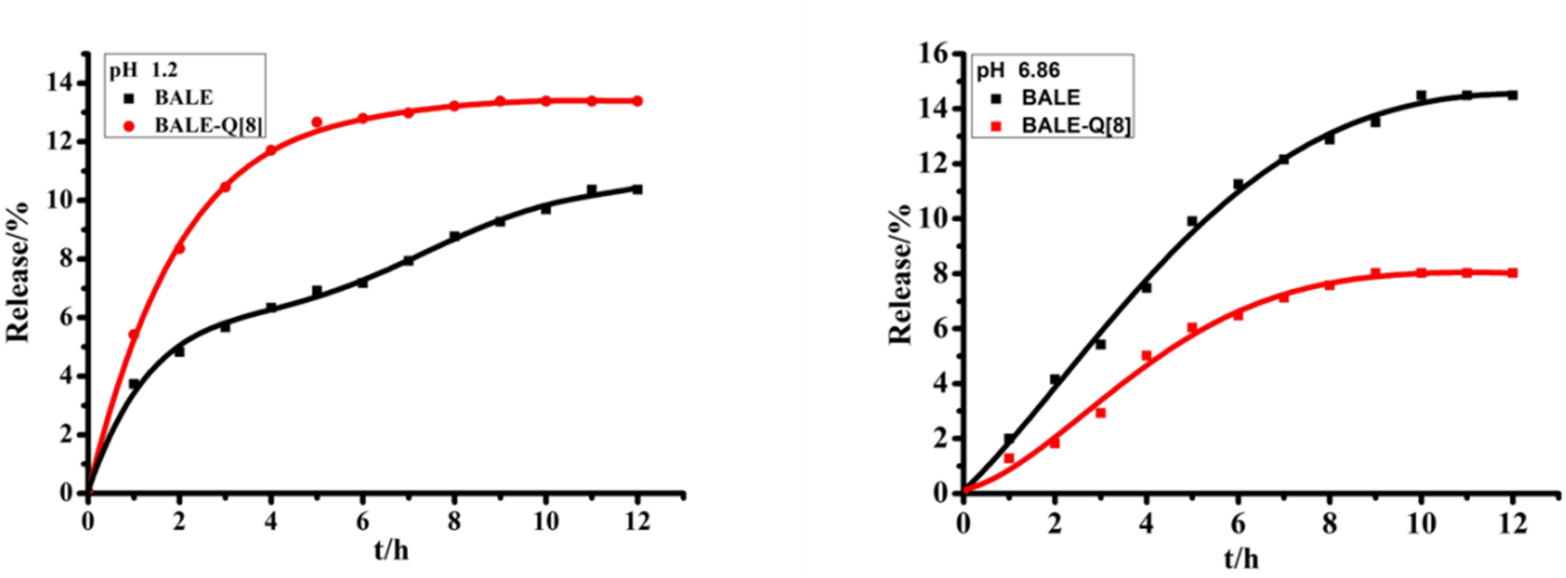
The release curves of BALE and BALE–Q[8].

In vitro release studies were performed according to [43–44]. As a result of the solubility of BALE in water being extremely poor, BALE in artificial gastric juice (pH 1.2, hydrochloric acid solution) and artificial intestinal juice (pH 6.8, phosphate buffer solution) can be detected. After 12 h, the degree of release was 11.26% (BALE) and 13.39% (BALE–Q[8]), respectively in artificial gastric juice and 14.49% (BALE) and 8.02% (BALE–Q[8]), respectively in artificial intestinal juice. In addition, the release and degradation tended to reach an equilibrium (Figure 10). The release rates of the BALE–Q[8] complex was slower than that of BALE in artificial intestinal juice, but it was faster than the BALE in artificial gastric juice.

When comparing the interactions of chrysin [45] and baicalin [46] with Q[8] previously reported by our group, they showed different interaction models. Chrysin is totally inserted into the cavity of Q[8], and due to the fact that the hydroxy groups of the glucoside of baicalin and Q[8] fare forming hydrogen bonds, the cycle B and and a part of cycle C of baicalin inserted into the cavity of the Q[8]. This may be attributed to chrysin containing one hydroxy group less than baicalein, which reduced the hydrophilicity of cycle A, making it enter into the hydrophobic cavity of Q[8] easily. Because the hydroxy group on the carboxylic acid of the baicalin formed a hydrogen bond with the oxygen atoms of Q[8] at the portal, cycle C of baicalin was pushed into the cavity of Q[8]. Therefore, the inclusion model of cucurbit[8] with flavonoid compounds was determined by the structure of the specific flavonoid.

## Conclusion

In this study, our results showed that BALE formed an inclusion complex (1:1) with cucurbit[8]uril. The inclusion constants of BALE with Q[8] obtained from the UV–vis absorption data was 2.15 × 10^7^ L·mol^−1^ (10 mol·L^−1^ HCl), 3.59 × 10^7^ L·mol^−1^ (10^−2^ mol·L^−1^ HCl) and 3.4 × 10^5^ L·mol^−1^ (neutral water). The solubility of BALE increased 4.67-fold in the phase-solubility experiment when the concentration of Q[8] was 1 × 10^−4^ mol·L^−1^. A study of the UV–vis absorption spectra with time showed that Q[8] significantly increased the stability of BALE. The antioxidant activity of BALE–Q[8] was investigated using the ABTS^+·^ method. The BALE–Q[8] inclusion complex had no significant influence on the scavenging effect toward ABTS^+·^ radicals when compared to BALE; the IC_50_ vales were 1.87 × 10^−6^ mol·L^−1^ and 2.14 × 10^−6^ mol·L^−1^, respectively. In vitro release studies have shown that the release rates of the BALE–Q[8] complex is slower than that of BALE in artificial intestinal juice, but it is faster than BALE in artificial gastric juice. Our results provide a new approach and theoretical basis for the development and utilization of baicalein.

## Supporting Information

File 1 Instrumentation, materials and methods.

File 2 NMR data.
