# Supplementary material for: The interaction between cucurbit[8]uril and baicalein and the effect on baicalein properties
Source: Beilstein J Org Chem. 2020 Jan 10;16:71–7. doi: 10.3762/bjoc.16.9 (PMC6964663; doi:10.3762/bjoc.16.9)
Supplement: File 1 — Instrumentation, materials and methods. [file Beilstein_J_Org_Chem-16-71-s001.pdf]

BEILSTEIN JOURNAL OF ORGANIC CHEMISTRY

Supporting Information

for

The interaction between cucurbit[8]uril and baicalein and the effect on baicalein properties

Xiaodong Zhang, Jun Xie, Zhiling Xu, Zhu Tao and Qianjun Zhang

Beilstein J. Org. Chem. **2020,** *16,* 71–77. doi:10.3762/bjoc.16.9

Instrumentation, materials and methods

License and Terms: This is a supporting information file under the terms of the Creative Commons Attribution License (https://creativecommons.org/licenses/by/4.0). Please note that the reuse, redistribution and reproduction in particular requires that the authors and source are credited.
The license is subject to the *Beilstein Journal of Organic Chemistry* terms and conditions: (https://www.beilstein-journals.org/bjoc)

Table of contents

Instrumentation S2

Materials S2

Methods S2

^{1}H NMR spectroscopy S2

UV–vis spectroscopy S3

IR spectroscopy and DTA S4

Stability study S4

Solubility study (phase solubility method) S4

ABTS radical-scavenging activity S5

In vitro release studies S5

Mass Spectrum S6

References S6

Instrumentation

TU-1901 double beam UV-visible spectrophotometer; RE-52A rotary evaporator; VERTEX70 (Bruker, Germany)Fourier infrared spectrometer; FA2204N electronic balance; The German resistance chi STA449C thermal analyzer; JNM-ECZ400s MHz nuclear magnetic resonance (NMR) spectrometer, Agilent 6545 Q-TOF.

Materials

Q[8] was prepared in the Key Laboratory of Macrocyclic and Supramolecular Chemistry of Guizhou province (purity≥98%); baicalein (purity ≥98%) and ABTS were purchased from Aladdin.

BALE–Q[8]: A certain amount of Q[8] and BALE were weighed according to a 1:1 molar ratio of NQ[8]/NBALE, dissolved with distilled water and methanol, and stirred for 1 h. The solvent was removed in vacuo to obtain the BALE–Q[8]inclusion compound (1:1).

Methods

^{1}H NMR spectroscopy

To study the host–guest complexation of Q[8] and BALE, 1×10^{-6} mol of Q[8], BALE and BALE–Q[8] in 5×10^{-4} L DCl (10 mol·L^{-1}) with little 3-(trimethylsilyl)propionic-2,2,3,3,-d_4 acid were prepared and their corresponding ^{1}H NMR spectra and ^{1}H NOESY were recorded at 20 °C on a JNM-ECZ400s MHz nuclear magnetic resonance (NMR) spectrometer.

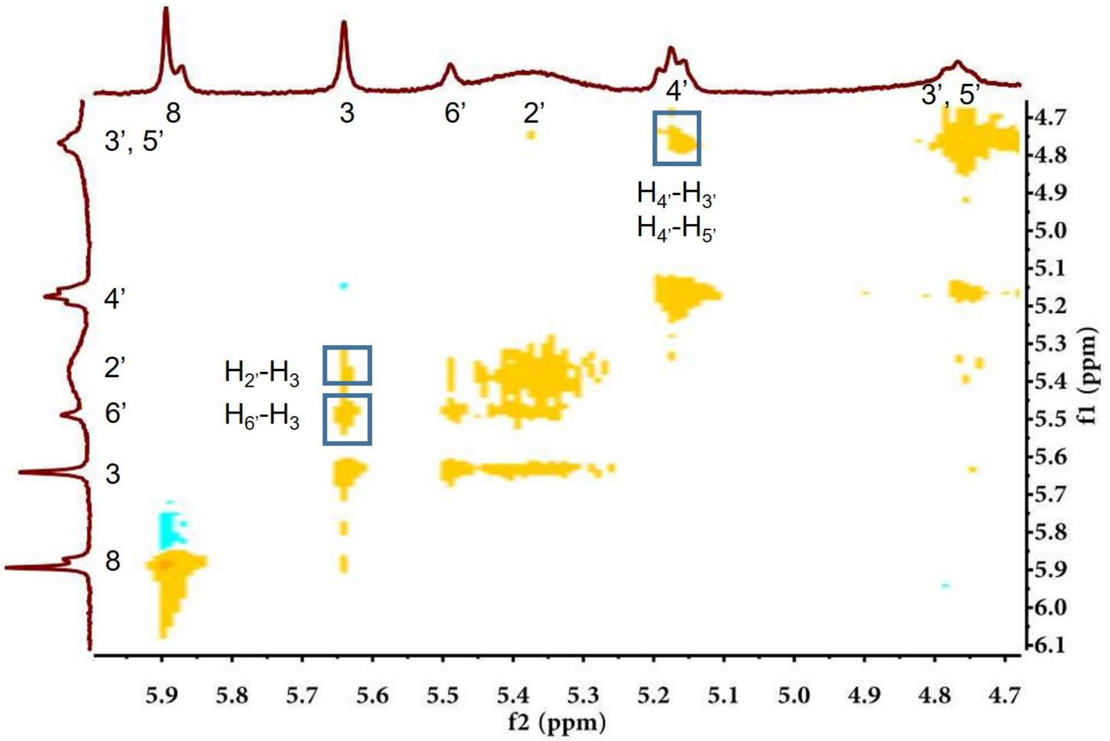

Figure S1: NOESY spectrum (400 MHz) of the BALE–Q[8] inclusion complex recorded in DCl.

UV–vis spectroscopy

The UV absorption spectra of the host–guest complexes were recorded on a TU-1901 double beam UV–visible spectrophotometer at room temperature. A methanol solution of BALE was prepared at a concentration of 1.00×10^{-3} mol·L^{-1} and an aqueous solution of Q[8] was prepared at a concentration of 1.00×10^{-4} mol·L^{-1} to determine their absorption spectra. Samples of these solutions (including 2% methanol) were combined to give solutions with a BALE:Q[8] ratio of 0, 0.2, 0.4......and 3.0. The formation constants of the BALE–Q[8] complexes (K) (1:1) were calculated according to curve fitting methods using Job's method fixing the system concentration (2×10^{-5} mol·L^{-1}) and molar ratio of NQ[8]:N(Q[8] + BALE) = 0, 0.1, 0.2, 0.3, ..., 1.0.

IR spectroscopy and DTA

BALE, Q[8], a physical mixture of BALE and Q[8] (NQ[8]:NBALE= 1:1)and BALE–Q[8] were weighed, respectively. KBr was added to prepare KBr discs of the samples to record the IR spectra over a wavenumber range of 4000–500 cm^{-1}. In addition, 10 mg of each sample was weighed for DTA.

Stability study

Aqueous solutions of BALE and BALE–Q[8] were prepared at a concentration of 2×10^{-5} $mol \cdot L^{-1}$ and placed in air. The solutions were sampled over time at room temperature and their UV absorption intensity was measured. The decomposition rate constants of BALE were obtained by plotting the absorbance values of BALE without decomposition and the stability was investigated.

Solubility study (phase solubility method) [1]

1. Calibration curve for the BALE standard solution

A methanol solution of BALE at a concentration of 1×10^{-3} $mol \cdot L^{-1}$ was used as a stock solution. Different volumes (0.02, 0.04, 0.06, 0.08 and 0.10 mL) of BALE were placed 10 mL volumetric flasks. The volume of the methanol was kept unchanged at 1%. The absorbance at λ_{270} nm was measured and the standard calibration curve of BALE was obtained.

2. Correction curve of the BALE standard solution in the presence of Q[8]

A methanol solution of BALE at a concentration of $1 \times 10^{-3}$$mol \cdot L^{-1}$ was used as the stock solution. Different volumes (0.02, 0.04, 0.06, 0.08 and 0.10 mL) of the BALE stock solution were added to 10 mL volumetric flasks and an aqueous solution of Q[8] was added. The volume was adjusted to 1% by volume of methanol. The calibration curve for BALE in the presence of Q[8] was obtained by measuring the absorbance at λ_{270} nm.

3. Determination of the solubility of BALE

A total of 5 mg of BALE was weighed into 25 mL volumetric flasks and different volumes (0, 5, 10, 15, 20 and 25 mL) of a 1×10^{-4} $mol \cdot L^{-1}$ Q[8]

solution added. Water was used to make up the remaining volume and the mixtures subjected to ultrasonication for 60 min until the solid-liquid balance. The absorbance was measured at λ_{270} nm after filtration through a 0.5 μm membrane filter. The BALE content was calculated according to the working curve.

ABTS radical-scavenging activity [2]

The antioxidant activities of the test compounds were evaluated on the basis of the radical-scavenging effect of the stable ABTS free radical. An $ABTS^{+\bullet}$ solution was prepared by mixing 7.0 mmol of ABTS salt with 2.45 mmol of potassium persulfate in 10 mL of distilled water. The solution was kept at room temperature in the dark for 24 h prior to use. Fresh $ABTS^{+\bullet}$ solutions were prepared for each assay. Different volumes of 1×10^{-3} $mol{\cdot}L^{-1}$ BALE or BALE@Q[8] solution was added into 10 mL volumetric flasks, using water to make up the remaining volume. Then, 0.1 mL of the $ABTS^{+\bullet}$ stock solution was added with stirring. Clearance time curve: The absorbance was measured at 415 nm every 0.5 min at room temperature. The percentage inhibition of the test compounds was calculated using the following equation:

$$I\ \%= [(A_{control} - A_{sample})/A_{control}] \times 100\%$$

Where $A_{control}$ is the absorbance of the control reaction (containing all the reagents except the test compound) and $A_{samples}$ (the absorbance of the reaction containing the test compound). Clearance rate curve: The samples were left to stand still for 30 min, the absorbance at different concentrations was measured at 415 nm at room temperature and the half inhibition concentration (IC_{50}) was calculated using the clearance rate curve.

In vitro release studies [3,4]

The in vitro drug release behavior of the inclusion compound was investigated using a constant temperature shaking method. After accurately weighing 0.01 mmol of BALE and 0.01 mmol of BALE–Q[8], the samples were added to dialysis bags and placed in a thermostatic shaker containing artificial

intestinal fluid (pH = 6.8 phosphate buffer solution, 500 mL) or artificial gastric juice (pH = 1.2 hydrochloric acid solution, 500 mL), shaken slowly in a water bath at 37 °C. At appropriate time intervals, 3 mL of each sample was removed, adding the same volume of fresh release medium at the same time. The absorbance of the samples were measured at 270 nm, the release rate of BALE calculated using the absorbance of BALE.

Mass spectrum

The ESI–TOF Mass Spectrometry of the BALE–Q[8] inclusion complex were recorded on an Agilent 6545 Q-TOF at room temperature. And an aqueous solution of BALE–Q[8] was prepared at a concentration of 1.00 × 10^{-4} mol·L^{-1}, then the solution was filtered and tested by MS.

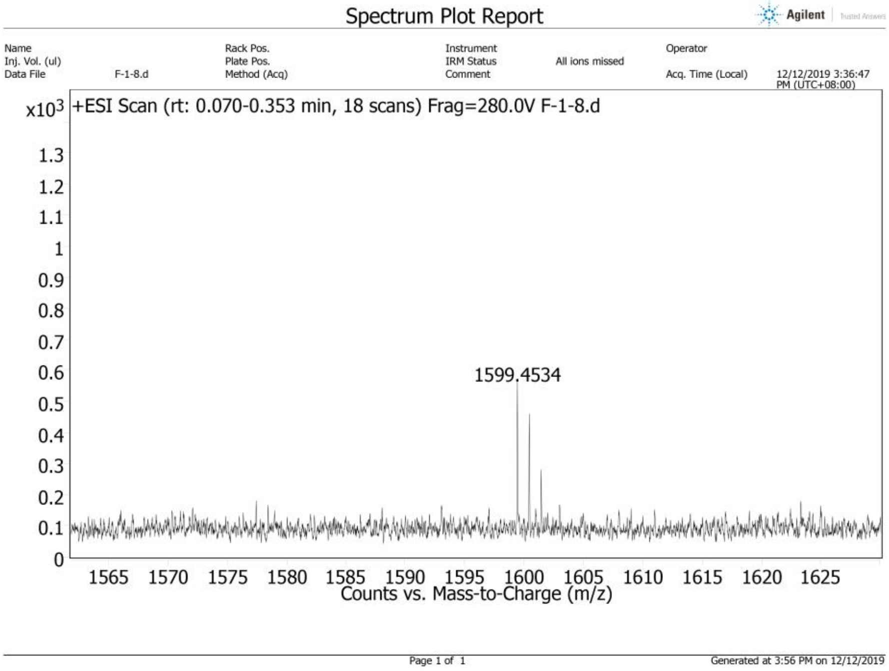

Figure S2: ESI–TOF mass spectrometry of the BALE–Q[8] inclusion complex

References

1. Alfonsi, R.; Attivi, D.; Astier, A.; Socha, M.; Morice, S.; Gibaud, S. *Ann. Pharm. Fr.* **2013,** 71(3), 186-192.

2. Lee, B. W.; Lee, J. H.; Gal, S. W.; Moon, Y. H.; Park, K. H. *Biosci. Biotechnol. Biochem.* **2006,** 70(2), 427-432.

3. *Chinese Pharmacopoeia Commission*, *Chinese Pharmacopoeia*, 2015, *Section Two*, China Medical Science and Technology Press,Beijing, **2015,** 85-86

4. Yang, C.; Wang, Z.; Ou, C.; Chen, M.; Wang, L.; Yang, Z. *Chem. Commun.* **2014,** 50(66), 9413-9415.
